# Transcriptional capacity limits copper resistance in yeast harboring highly expanded *CUP1* arrays

**DOI:** 10.17912/micropub.biology.002172

**Published:** 2026-05-14

**Authors:** Hiroaki Takesue, Satoshi Okada, Takashi Ito

**Affiliations:** 1 Department of Biochemistry, Kyushu University Graduate School of Medical Sciences, Fukuoka, Japan; 2 Present address: Division of Transcriptomics, Medical Institute of Bioregulation, Kyushu University, Fukuoka, Japan; 3 Present address: Department of Biological Science, Faculty of Science and Engineering, Yasuda Women's University, Hiroshima, Japan

## Abstract

Copper resistance in the budding yeast *Saccharomyces cerevisiae* is primarily mediated by the tandemly arrayed metallothionein gene *CUP1* . We analyzed eight isogenic strains harboring *CUP1* arrays of varying lengths, including those artificially expanded beyond their natural ranges. *CUP1* mRNA levels and copper resistance increased with copy number before reaching a plateau. Increased dosage of the transcriptional activator Cup2 partially mitigated the plateaued resistance in strains with intermediate, but not high, copy numbers. These findings indicate that *CUP1* confers resistance dose-dependently until transcriptional capacity becomes limiting, suggesting a possible strategy for engineering extreme copper resistance.

**Figure 1. Copper resistance of S. cerevisiae strains with different CUP1 copy numbers:** (A) Schematic representation of the *CUP1* array on chromosome VIII. Strains with varying copy numbers of the 2-kb repeat unit containing *CUP1* (CUP1RU) were analyzed. (B) Estimation of *CUP1* copy number by quantitative PCR (qPCR) and nanopore sequencing-based analysis (blastn). Eight strains with distinct *CUP1* copy numbers, designated as 1×, 2×, 5×, 14×, 26×, 80×, 250×, and 380×, were selected for further analysis. (C) *CUP1* mRNA levels. RT-qPCR analysis of *CUP1* expression in the eight strains described in (B). Cells were cultured in either YPD (left) or SC (right) medium, with or without the addition of 0.1 mM CuSO₄. *CUP1* mRNA levels were normalized to those of *ACT1* as an internal control. Solid lines represent relative *CUP1* mRNA levels under basal (orange) and copper-induced (blue) conditions. The green dashed line indicates the fold-induction of *CUP1* mRNA in response to copper. (D) Copper resistance of strains with different *CUP1* copy numbers. Growth (OD _620_ ) after 24 h in YPD (left) or SC (right) medium containing various concentrations of CuSO _4_ was normalized to growth in the medium without copper supplementation. CuSO _4_ was supplemented in 1.5 mM increments (0–15 mM) for YPD and 0.5 mM increments (0–5 mM) for SC medium. WT, wild-type. (E) Relationship between *CUP1* mRNA levels and copper resistance. IC _50_ values calculated from dose–response curves in YPD (left) or SC (right) medium were plotted against relative *CUP1* mRNA levels. Solid lines indicate linear regression. (F) Effect of increased *CUP2* dosage on copper resistance. Strains transformed with the YCpKanMX-CUP2 plasmid were cultured in YPD (left) or SC (right) medium supplemented with G418 (200 μg/mL) and various concentrations of CuSO _4_ . Relative growth was determined as described in (D). (G) Impact of increased *CUP2* dosage on IC _50_ . The differences in IC _50_ values between the presence ( *CUP2* (+)) and absence (WT) of the YCpKanMX-CUP2 plasmid (ΔIC _50_ = IC _50_ *^CUP2^* ^(+)^ − IC _50_^WT^ ) are shown for YPD and SC media.

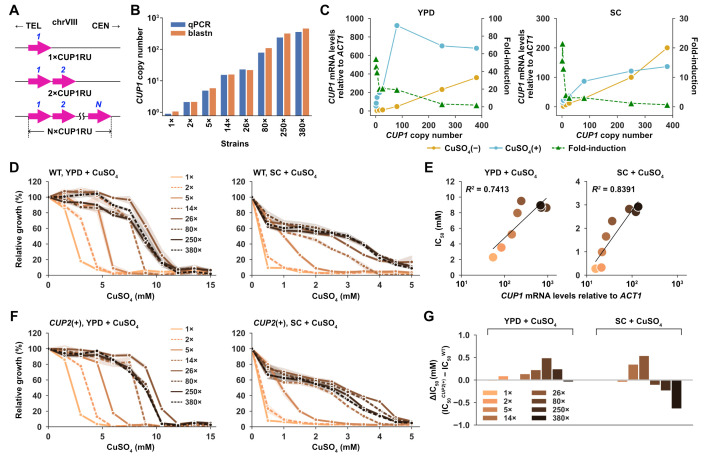



## Description

Copper is an essential metal for cell viability but becomes toxic in excess. Cells have thus developed elaborate systems to maintain copper homeostasis, including mechanisms for buffering the effects of environmental copper. Among such systems in the budding yeast *Saccharomyces cerevisiae* (Shi et al., 2021), the metallothionein Cup1 sequesters excess intracellular copper, thereby playing a central role in resistance (Fogel et al., 1983). The expression of *CUP1* gene is induced by copper via the action of the transcriptional activator Cup2 (Welch et al., 1989). The *CUP1* gene often forms a tandem array, with a repeat unit size ranging from 1.2 to 2.0 kb among different strains (Zhao et al., 2014). The copy number comprising this array varies from 0 to 79 among natural isolates (Crosato et al., 2020). It has been shown that strains with high *CUP1* copy numbers are generally more resistant to copper than those with lower copy numbers. However, this correlation is not always robust; *CUP1* copy number variation alone was reported to explain 44.5% of the phenotypic variation (Peter et al., 2018). The involvement of other loci, such as *SSU1* encoding a sulfite efflux pump, has also been demonstrated (Crosato et al., 2020; Onetto et al., 2023). Previous studies on the relationship between *CUP1* copy number and copper resistance have used natural isolates with variable copy numbers, which inevitably possess different genetic backgrounds, confounding the interpretation of the results. To precisely evaluate the effect of *CUP1* copy number on copper resistance, it is ideal to use isogenic strains spanning a wide range of *CUP1* array lengths. Yet the lack of tools for modulating array length has rendered such a straightforward approach elusive.

We recently developed a Cas9 nickase-based method for tandem gene array expansion termed break-induced replication-mediated tandem repeat expansion (BITREx) (Takesue et al., 2025). We successfully applied BITREx to expand the *CUP1* array in a standard laboratory strain from 14 to over 500 copies *in situ* without detectable chromosomal abnormalities; notably, this massive expansion was achieved in the absence of any copper-related selection pressure. We also showed that nicotinamide can contract the *CUP1* array, particularly when bound by catalytically inactive Cas9 (Doi et al., 2021; Takesue et al., 2025). Using these approaches, we prepared a set of eight isogenic strains with *CUP1* copy numbers ranging from 1 to ~380, including those harboring extremely long arrays artificially expanded beyond natural ranges (Figures 1A and 1B). This study exploits these strains to examine the effect of *CUP1* copy number on copper resistance.

We first measured *CUP1* mRNA levels in the eight strains by RT-qPCR under both basal and copper-induced conditions ( Figure 1C ). Basal *CUP1* mRNA levels generally correlated with copy number in both yeast extract–peptone–dextrose (YPD) and synthetic complete (SC) media. However, induced *CUP1* mRNA levels increased with copy number but reached a plateau in high-copy strains. Accordingly, the fold-induction of *CUP1* mRNA by copper declined as the copy number increased.

We next assessed the copper resistance of these strains by monitoring their growth as optical density at 620 nm (OD _620_ ) in media containing increasing concentrations of CuSO _4_ ( Figure 1D ). Growth after 24 h at each copper concentration was normalized to that in the medium without copper supplementation. Copper resistance increased with *CUP1* copy number but reached a plateau; the half-maximal inhibitory concentration (IC _50_ ) values—derived from dose–response curves—plateaued in the 26× and 80× strains in YPD and SC media, respectively ( Figure 1E ).

To examine the relationship between copper resistance and *CUP1* expression, we plotted the IC _50_ values against the induced *CUP1* mRNA levels ( Figure 1E ). Although the range of IC _50_ values was wider and higher in YPD medium than in SC medium, a strong positive correlation was observed between *CUP1* mRNA levels and copper resistance in both media.

The plateau in induced *CUP1* mRNA levels suggested that the transcriptional activator Cup2, which mediates copper-induced activation, may become limiting. This would leave a significant fraction of *CUP1* promoters unoccupied in high-copy-number strains. Given the relationship between *CUP1* mRNA levels and copper resistance, we hypothesized that supplementing Cup2 could overcome the plateau effect in resistance. To test this, we examined the impact of increased *CUP2* dosage by introducing a centromeric plasmid carrying the *CUP2* gene ( Figure 1F ). The results showed that the increased *CUP2* dosage improved resistance, particularly in strains with intermediate copy numbers, but had limited or even adverse effects in strains with extremely high copy numbers. Consequently, the difference of IC _50_ values with and without the *CUP2* plasmid (ΔIC _50_ ) followed a convex relationship relative to *CUP1* copy number, with the maximal increase in resistance observed in the 80× strain in YPD medium and the 26× strain in SC medium ( Figure 1G ).

Together, these findings indicate that *CUP1* array expansion confers copper resistance in a dose-dependent manner until transcriptional capacity becomes limiting. Furthermore, our results suggest a potential strategy for engineering strains with extreme copper resistance through the coordinated optimization of gene copy number and transcriptional activator dosage. Intriguingly, a previous study reported that a clone derived from a natural copper-resistant isolate with large-scale chromosomal rearrangements was trisomic for the chromosome VIII segment encoding the *CUP1* array and disomic for the chromosome VII segment containing *CUP2* . The resistance of that strain was shown to be dependent on the increased dosage of *CUP2* , thereby bolstering the biological relevance of our findings (Chang et al., 2013).

## Methods

*Plasmid construction*

The centromeric plasmid carrying *CUP2* and *KanMX6* (YCpKanMX6-CUP2) was constructed via seamless cloning using the NEBuilder HiFi DNA Assembly kit (New England Biolabs) and then transformed into *E. coli* competent cell, Champion ^TM^ DH5α high (SMOBIO). The cloned *CUP2* fragment includes its own promoter and terminator (Chromosome VII: 190,469–191,977).

*Yeast strains*

All yeast strain used in this study were derived from BY4741 ( *MAT* **a** *his3* Δ1 *leu2* Δ0 *met15* Δ0 *ura3* Δ0) (Brachmann et al., 1998).

*Yeast growth and copper resistance assay*

Yeast cells were grown at 30°C overnight in 125 µL of the YPD medium or SC medium supplemented with 2% glucose. The optical density at 620 nm (OD _620_ ) was measured on the following day using Absorbance 96 Plate Reader (Byonoy). Cultures were then adequately diluted and inoculated into 125 µL of fresh medium containing various concentrations of CuSO _4_ (0–15 mM). OD _620_ was recorded after 24 h of incubation and normalized to the growth in the medium without copper supplementation (0 mM CuSO _4_ ).

*Genomic DNA extraction*

For qPCR analysis, genomic DNA was extracted using the GC prep method (Blount et al., 2016). For nanopore sequencing, high-molecular-weight genomic DNA was extracted using the Monarch HMW DNA Extraction Kit for Tissue (New England Biolabs). To minimize DNA fragmentation, vortexing was avoided, and mixing was performed via gentle pipetting with wide-bore tips, as described previously (Takesue et al., 2025).

*RNA extraction and cDNA synthesis*

Total RNA was extracted from up to 1×10 ^8^ cells using the Quick-RNA Fungal/Bacterial Miniprep Kit (Zymo Research) according to the manufacturer’s instructions. RNA concentration was determined using a Qubit 2.0 Fluorometer with the Qubit RNA BR Assay System (Thermo Fisher Scientific). Subsequently, 800 ng of total RNA was reverse-transcribed using random primers and the High-Capacity cDNA Reverse Transcription Kit (Thermo Fisher Scientific).

*Quantitative PCR (qPCR)*

Genomic DNA or cDNA was diluted 10-fold with distilled water prior to qPCR. Each reaction (20 μL) contained 2 μL of diluted DNA, 10 μL of KOD SYBR qPCR Mix (TOYOBO), 0.04 μL of 50× ROX Reference Dye (TOYOBO), and 2 pmol each of the forward and reverse primers (listed below). qPCR was performed in duplicate using the QuantStudio 3 Real-Time PCR System (Applied Biosystems). The thermal cycling conditions were as follows: initial denaturation at 98°C for 2 min, followed by 40 cycles of 98°C for 10 s, 55°C for 10 s, and 68°C for 30 s. Standard curves were generated for each run using 10-fold serial dilutions. *CUP1* levels were normalized to *ACT1* . The *CUP1* copy number in the standard curves was calibrated based on nanopore sequencing results of the BY4741 strain.

*Nanopore sequencing*

DNA libraries for whole-genome sequencing were prepared using the Ligation Sequencing Kit (SQK-LSK114, Oxford Nanopore Technologies) and the Native Barcoding Kit (SQK-NBD114, Oxford Nanopore Technologies). The manufacturer's protocol was modified to preserve DNA integrity and improve efficiency: DNA fragmentation was omitted; enzymatic repair (20°C and 65°C) and ligation steps were extended to 30 min each; and the elution time with 0.4× AMPure XP beads was extended to 20 min. Libraries were sequenced on a PromethION 2 Solo sequencer using FLO-PRO114M (R10.4.1) flow cells. MinKNOW software was used for device control, with a run time of 72 h. Basecalling was performed using Dorado v0.7.3, and data quality was assessed using NanoPlot (https://github.com/wdecoster/NanoPlot). All raw sequencing data were deposited with links to BioProject PRJDB40683 in the DDBJ BioProject database.

To accurately estimate the repeat unit number while minimizing the impact of read clipping, all reads containing the repeat unit were collected using Minimap2 (https://github.com/lh3/minimap2). Subsequently, the *CUP1* reference sequence was used as a query for BLAST searches against the collected reads. The copy number was estimated based on the number of BLAST hits, as described previously (Takesue et al., 2025). The analysis pipeline for tandem gene arrays is publicly available at Zenodo (https://zenodo.org/records/18440611).

*Generative AI and AI-assisted technologies*

During the preparation of this work, the authors used Gemini 3 Flash to improve the readability of certain sentences. After using this tool/service, the authors reviewed and edited the content as needed and take full responsibility for the content of the publication.

## Reagents

The yeast strains used in this study.

**Table:** 

Strain	Genotype	Available from
YIT10556	*1* × *CUP1RU pFA6a-pCUP2-yGEV-tADH1-HphMX(MfeI-cut@pCUP2) YIplac128-pGAL1-Cas9(D10A)-tADH1(AgeI-cut@pGAL1)*	Ito Lab
YIT10557	2× *CUP1RU pFA6a-pCUP2-yGEV-tADH1-HphMX(MfeI-cut@pCUP2) YIplac128-pGAL1-Cas9(D10A)-tADH1(AgeI-cut@pGAL1)*	Ito Lab
YIT10559	*5×CUP1RU pFA6a-pCUP2-yGEV-tADH1-HphMX(MfeI-cut@pCUP2) YIplac128-pGAL1-Cas9(D10A)-tADH1(AgeI-cut@pGAL1)*	Ito Lab
YIT8035	*14* × *CUP1RU pFA6a-pCUP2-yGEV-tADH1-HphMX(MfeI-cut@pCUP2) YIplac128-pGAL1-Cas9(D10A)-tADH1(AgeI-cut@pGAL1)*	Ito Lab
YIT10364	*26* × *CUP1RU pFA6a-pCUP2-yGEV-tADH1-HphMX(MfeI-cut@pCUP2) YIplac128-pGAL1-Cas9(D10A)-tADH1(AgeI-cut@pGAL1)*	Ito Lab
YIT10368	*80×CUP1RU pFA6a-pCUP2-yGEV-tADH1-HphMX(MfeI-cut@pCUP2) YIplac128-pGAL1-Cas9(D10A)-tADH1(AgeI-cut@pGAL1)*	Ito Lab
YIT10652	*250×CUP1RU pFA6a-pCUP2-yGEV-tADH1-HphMX(MfeI-cut@pCUP2) YIplac128-pGAL1-Cas9(D10A)-tADH1(AgeI-cut@pGAL1)*	Ito Lab
YIT11126	*380×CUP1RU pFA6a-pCUP2-yGEV-tADH1-HphMX(MfeI-cut@pCUP2) YIplac128-pGAL1-Cas9(D10A)-tADH1(AgeI-cut@pGAL1)*	Ito Lab

The plasmid used in this study.

**Table:** 

Plasmid	Genotype	Description
59-9	YCpKanMX6-CUP2	A centromeric plasmid harboring *CUP2* and the *KanMX6* selection marker.

The PCR primers used in this study.

**Table:** 

Name	Sequence	Description	Reference
*ACT1* -F	CGCTGCTCAATCTTCTTCAA	*ACT1* quantification by qPCR/RT-qPCR	Takesue et al., 2025
*ACT1* -R	GTAGTTTGGTCAATACCGGC	*ACT1* quantification by qPCR/RT-qPCR	Takesue et al., 2025
*CUP1* -F	TTCGTTTCATTTCCCAGAGC	*CUP1* quantification by qPCR/RT-qPCR	Takesue et al., 2025
*CUP1* -R	CAATGCCAATGTGGTAGCTG	*CUP1* quantification by qPCR/RT-qPCR	Takesue et al., 2025
